# Pathologic fracture does not influence prognosis in stage IIB osteosarcoma: a case–control study

**DOI:** 10.1186/1477-7819-11-148

**Published:** 2013-06-24

**Authors:** Dongqing Zuo, Longpo Zheng, Wei Sun, Yingqi Hua, Zhengdong Cai

**Affiliations:** 1 Musculoskeletal Oncology Center, Shanghai 10th People's Hospital, Tongji University School of Medicine, Shanghai, 200072, China

**Keywords:** Amputation, Disease-free Survival, Limb Salvage, Osteosarcoma, Pathologic Fracture

## Abstract

**Objective:**

This study tested the implication of pathologic fractures on the prognosis in stage IIb osteosarcoma.

**Methods:**

A single center retrospective evaluation of clinical management and oncologic outcome was conducted with 15 pathological fracture patients (M:F = 10:5; age: mean 23.2, range 12–42) and 50 non-fracture patients between April 2002 and December 2010. These stage IIB osteosarcoma patients were matched for age, tumor site (femur, tibia, and humerus), and osteosarcoma subtype (i.e., control patients with osteosarcoma in the same sites as the fracture patients). All osteosarcoma patients with pathological fractures underwent brace or cast immobilization, adjuvant chemotherapy, and limb salvage surgery or amputation. Musculoskeletal Tumor Society (MSTS) functional scores were assessed. The mean follow-up time was 34.7 months (range, 8–47 months).

**Results:**

Following limb salvage surgery, no statistical differences were observed in major complications (fracture = 20.0%, control = 12.0%, *P* = 0.43) or local recurrence complications (fracture = 26.7%, control = 14.0%, *P* = 0.25). Overall 3-year survival rates of the fracture and control groups (66.7% and 75.3%, respectively) were not statistically different (*P* = 0.5190). Three-year disease-free survival rates of the fracture and control groups were 53.3% and 66.5%, respectively (*P* = 0.25).

**Conclusions:**

Pathologic fracture was not a prognostic indicator of recurrence or overall survival in localized osteosarcoma patients. Limb salvage can be achieved by and maintaining adequate surgical margins and applying adjuvant chemotherapy.

## Background

Pathologic fracture occurs in as many as 5% to 10% of all osteosarcoma patients, affecting patients both at the time of diagnosis and during chemotherapy treatment [1-3]. In the past, pathologic fracture of the extremities caused by localized osteosarcoma has been considered an absolute indication for amputation of the affected limb [1,4]. Recent advances in multi-agent adjuvant chemotherapy, surgical techniques, and radiographic imaging have not only dramatically improved overall prognosis in these patients, but also made limb salvage surgeries feasible [5]. Contemporary applications of such conservative treatments, however, have sparked controversy about the actual impact of these techniques on patient outcomes, including function independence and quality of life.

Amputation procedures have been traditionally recommended for osteosarcoma patients exhibiting pathological fractures based on the theory that fracture-induced hematomas increase the risk of unexpected micro-metastasis [6-8]. This widely published theory resulted in the observation that pathological fracture occurrence was directly and significantly linked with prognosis and, more specifically, with the higher mortality rates related to metastatic cancer progression [9]. Several recent studies have, however, demonstrated that pathological fracture has no prognostic significance in patients with high-grade extremity osteosarcoma [10,11]. Such fractures are also poor indictors of local recurrence risk, though an association with increased mortality rate has been reported [12]. Conversely, a multi-center study conducted through a cooperative effort of the Musculoskeletal Tumor Society (MSTS) revealed findings supporting the use of pathologic fracture as an indicator of local recurrence and mortality risks in osteosarcoma patients [5]. Thus, a significant controversy pertaining to the usefulness of pathological fracture as a prognostic indicator of both mortality and recurrence has appeared in current scientific literature.

The body of published work examining pathological fracture in osteosarcoma patients has grown in recent decades, including numerous single center [10-19] and multi-center studies [5]. Unfortunately, the relative rarity of osteosarcoma patients has commonly resulted in inadequate matching of experimental and control subjects, with most studies including patients matched for cancer stage only. Because these study designs conduct comparisons without considering important parameters, such as patient age, classification, and lesion site, broad application of these findings is not possible. Furthermore, few of these studies have directly reported the occurrence of pathological fracture cases with appropriately matched controls. These omissions may be a primary contributor to the discrepancy between reports of the prognostic value of pathological fracture in these patients.

While the prognostic value of pathological fractures remains a subject of intense debate, proper surgical treatment for these fractures is equally controversial. Limb salvage, avoiding amputation, has been recently linked with direct benefits in several psychological factors and total quality of life. Psychometric parameters were significantly improved in limb salvage patients compared to amputees, and limb salvage patients exhibited improved material well being through increased success in occupational relations, creative-aesthetic behaviors, and sports activities [20]. In order to avoid amputation and thus preserve patient quality of life for osteosarcoma patients, improving conservative limb salvage strategies is of great clinical importance.

Only in recent decades have contemporary treatments applying improved surgical technologies achieved significant success in salvaging extremities affected by pathological osteosarcoma patients. In fact, modern treatment centers may offer limb salvage surgeries over amputation to as many as 80% of osteosarcoma patients [21]. Using adequate margins of excision, limb-sparing results can be achieved without undue risk of metastatic events, though surgeons still face the significant challenge of controlling the elevated rate of local recurrence in affected extremities [13]. One study even reports that limb salvage patients still exhibited better survival compared to amputees even when local recurrence occurred, in part due to more effective treatments for these localized occurrences. Additionally, the same study reported that fewer than 10% of limb salvage patients exhibited cancer metastasis [21]. Despite these positive indicators for the effectiveness of limb salvage surgery combined with chemotherapy, many treatment centers may still overuse amputation treatments in cases where limb salvage is possible.

The current study was designed to assess the prognostic value of pathologic fracture in localized osteosarcoma patients. Additionally, the efficacy of the two surgical options still widely employed in clinical practice, amputation and limb salvage, were explored. The findings provide clinicians and researchers with a better understanding of the benefits and limitations of these surgical alternatives for both improving patient functional outcomes and reducing mortality risks.

## Methods

### Study design and patient selection

A retrospective survey of 15 patients (M:F = 10:5; age: mean 23.2 years, range 12–42 years) with localized primary osteosarcoma complicated by pathologic fracture of the extremities (fracture group) and 50 patients with osteosarcoma without fractures (non-fracture group) was conducted at the Changhai Hospital of the Second Military Medical University (Shanghai) between April 2002 and December 2010 and written informed consent was obtained from the patient for publication of this report and any accompanying images. Patients of the fracture and non-fracture groups were matched for age, tumor stage, lesion site, and osteosarcoma subtype. The non-fracture group was used as a control group for evaluation of pathologic fracture as a prognostic indicator and for evaluation of surgical treatment efficacy between the two groups. Since the pathological fractures in osteosarcoma patients were diagnosed in proximal femur, distal femoral, and proximal humerus, we chose the control patients with osteosarcoma in these sites to match the fracture patients (Tables 1 and 2).

**Table 1:** Demographic and characteristic data for 15 cases of pathological fracture associated with stage IIB osteosarcoma

**No.**	**Gender**	**Age (year)**	**Site**	**Stage**	**P type**	**Surgery**	**Blood loss (mL)**	**Displacement**	**LR**	**Metastasis**	**CPL**	**RC type**	**FP time (months)**	**DF time**	**Tumor N stage**	**Outcome**
1	Male	12	P tibia	II B	classic	LBS	800	no	LR	+		pros	8	7	good	dead
2	Female	15	P femur	II B	p	LBS	850	no				pros	45	45	good	
3	Female	24	P femur	II B	classic	AMP	615	yes					39	39	poor	
4	Male	18	P humerus	II B	classic	LBS	705	yes				pros	46	46	good	
5	Female	34	D femur	II B	classic	AMP	868	yes					44	44	poor	
6	Male	19	D femur	II B	classic	LBS	920	no			lr	pros	39	39	good	
7	Male	20	P femur	II B	classic	LBS	1100	no				pros	45	45	good	
8	Female	35	P humerus	II B	classic	AMP	780	yes	LR	+			29	24	poor	dead
9	Female	22	D femur	II B	classic	LBS	850	no			infct	pros	47	47	good	
10	Female	19	P humerus	II B	classic	LBS	900	no				pros	23	22	poor	dead
11	Male	24	D femur	II B	classic	LBS	1000	no	LR			pros	46	36	good	
12	Male	42	P humerus	II B	classic	AMP	880	no					40	38	poor	dead
13	Male	22	D femur	II B	classic	LBS	780	no		+	brk	pros	18	16	poor	dead
14	Male	18	P humerus	II B	classic	LBS	1000	yes	LR			pros	39	34	good	
15	Female	23	D tibia	II B	classic	AMP	940	yes		+			12	11	poor	dead
Sum/Mean		23.2					865.9		4	4	3		34.7	32.9		6

P type: Pathologic type; classic: Classic osteosarcoma; p: Periosteal osteosarcoma; P: Proximal; D: Distal; AMP: Amputation; LBS: Limb salvage surgery; FP time: Follow-up time; CPL: Complication; RC type: Reconstruction type; infct: Infection; lr: Local recurrence; brk: Breakage; pros: Prosthesis.

**Table 2:** Clinical characteristics of the fracture group and non-fracture groups

	**Pathologic fracture patients (n = 15)**	**Patients without pathologic fracture (n = 50)**	***P *value (χ**^**2 **^**test)**
Age (mean)	23.2±8.0	21.8±12.2	0.69
Stage	IIB	IIB	
**Subtype**			0.36
Classic	93.3% (14)	98% (49)	
Others	6.7% (1)	2% (1)	
**Tumor site**			0.99
Femur	53.3% (8)	54% (27)	
Tibia	13.3% (2)	14% (7)	
Humerus	33.3% (5)	32% (16)	

Patients were included in the fracture group of the study based on the following: 1) presentation of isolated localized osteosarcoma lesion of the femur, humerus, or tibia; 2) diagnosis as high-grade osteosarcoma of classic subtype (stage Enneking IIB); and 3) no previous surgical treatment for pathological fracture. Notably, control patients were included based on the previous criteria 2 and 3, but not criteria 1. Patients excluded were those that presented with: 1) previous metastasis activity; 2) lesions in more than one extremity; 3) abnormal biopsy scar; 4) other cancer history or treatment; 5) tumor involvement of the neurovascular bundle; 6) multiple pulmonary metastases; and 7) contraindication of chemotherapy. These criteria were partly based on the recommendation of a review by Ruggieri et al. [22] for the surgical treatment of long bone pathological fractures. We also excluded those patients treated at other institutions.

In the fracture group, the most frequently observed histopathological subtype was conventional osteosarcoma (14/15, 93%). The most commonly involved sites were the distal femur (5/15, 33%) and the proximal humerus (5/15, 33%), followed by the proximal femur (3/15, 20%). Tumor stage was determined as stage IIB in all patients (15/15, 100%) according to the MSTS Staging System (Enneking 1986) using local radiography, computed tomography (CT) of the chest, and magnetic resonance imaging (MRI) in the most recent cases. Of these 15 patients, pathologic fracture was present in 8 patients (53%) at the time of diagnosis and occurred in 7 patients (47%) during chemotherapy. Demographic and clinical data of the fracture group is detailed listed in Table 1.

### Clinical management and assessment

Each of the 15 patients of the fracture group was immobilized by standard brace or plaster cast, according to the method of Scully et al. [5,19]. Patients were then followed for a minimum of 4 preoperative adjuvant chemotherapy cycles, according to the National Comprehensive Cancer Network (NCCN). In consideration of the patients’ clinical manifestation, tolerance situation, and the NCCN protocols, some first-line chemotherapeutic drugs were administrated preoperatively for 4–6 cycles in our hospital: methotrexate 60 g/m^2^, adriamycin 420 mg/m^2^, cisplatin 600 mg/m^2^, and standard-dose ifosfamide 30 g/m^2^.

Chemotherapy-related tumor necrosis rates were assessed by a senior pathologist, according to the method of Bacci et al. [23]: good response = tumor necrosis >90%, and poor response = tumor necrosis < 90%. The patients were assessed by imaging RECIST evaluation criteria, and tumors exhibiting partial response and above were considered as indication of limb salvage. Postoperative chemotherapy was determined by neoadjuvant chemotherapy assessment, tumor necrosis rate, and pathologic type. Furthermore, combined methotrexate and adriamycin in chemotherapeutic regimens was avoided due to severe hepatotoxicity.

### Limb salvage and amputation surgeries

Patients who responded well to chemotherapy treatment (10/15, 66%) with good symptom relief, fracture healing, tumor ossification, and increased bone formation upon radiographic examination were determined to be eligible for limb salvage surgery. Signs for limb salvage included: 1) large functional muscles since the resection of such muscles would make reconstruction difficult and functional loss; 2) good chemotherapeutic response because if chemotherapy could effectively eradicate the tumors, limb salvage would be of more significance; 3) no distant signs of metastasis since these would have made the reconstruction surgery meaningless. Reconstruction type was based on patients’ willingness, lesion location and surgical considerations (i.e., margins). The reconstruction types included: semi-shoulder prostheses (3/15, 20%), rotational hinged knee prosthesis systems (5/15, 33%), and proximal femur defect prostheses (2/15, 13%). Notably, personal preference of the patient was also considered when determining eligibility for limb salvage treatment.

Prosthesis reconstruction was applied in all cases of limb salvage surgery in the present study in order to reduce complication risks. Potential severe complications of limb salvage surgery included non-union at the graft-host junction, fatigue fracture, articular collapse, dislocation, degenerative joint disease, and failure of ligament attachments caused by allograft and allograft-prosthesis composite reconstruction techniques. Reconstruction of the distal femur was achieved using minimally invasive single medial incisions. We approached the incision of anterior-medialis of the quadriceps femoris (instead of bisecting it), to prevent the dysfunction of the extensor mechanism postsurgery.

The popliteal tendon and medial gastrocnemius were then transferred to cover the prosthesis and partially reconstruct the quadriceps region. Lesions of the distal humerus were reconstructed using modified Tikhoff-Linberg procedures [24]. Brachioradialis, pronator, and flexor carpi radialis muscles were sutured to the remaining biceps and triceps muscles to secure the soft tissues around the flared distal portion of the humeral prosthesis following prosthesis construction. Reconstruction of proximal femur fractures was achieved with proximal femur defect prosthesis combined with bone cement after resection of proximal femur. The remaining abductor was lowered to the proximal aspect of the prosthesis and attached to a metal loop with Dacron tape.

Surgical amputations of the affected extremity were conducted in five patients (33%) due to poor response to preoperative chemotherapy or insufficient surgical margins for limb salvage surgery. Above-knee amputations were performed in two patients (13%), forequarter amputation was performed in one patient (7%), and one patient (7%) underwent below-knee amputation.

Intraoperative tumor biopsies were taken from each patient from further laboratory analysis. All patients were subsequently transferred to the intensive care unit and closely observed for 24 hours following either amputation or limb salvage procedures. All patients underwent chemotherapy 3 weeks after surgery. Specialized rehabilitation schedules were proposed to aid in recovery of normal bodily functions. Notably, patients with fractures of the proximal tibia were immobilized for 3 weeks to ensure the stable connection of the extensor.

### Assessment of surgical parameters and postoperative outcomes

For patients of both the fracture and non-fracture (control) group, intraoperative blood loss and the surgical margin parameters (pseudocapsule rupture, and surgical margins) were recorded and analyzed. Surgical margins were assessed according to the system of Enneking et al. [25]. Wide margins of soft tissues were defined as tumor invaded mesooecium, which were determined by MRI. Osseous tissues were resected 3–4 cm away from the abnormal MRI signal area. Wide margin determination helped the resection. The borderline of the soft tumor tissues was obscure, and thus the suspicious tissues were resected along with the normal tissues. Additionally, biopsied tumor specimens were collected and assessed by senior pathologists in the current facility. The time taken for each patient to begin weight-bearing activities and walking were recorded.

All patients of both the fracture and non-fracture groups were observed in the outpatient clinic on an ongoing basis following discharge until either relapse or death was reported. General follow-up assessments included a physical examination, plain radiograph, CT scan, and bone scan (ECT). Bone scanning was performed to observe the existence of concentration phenomena in the bone area, which was used to position and observe the osteosarcoma recurrence after surgery. Usually, bone scanning was performed every 6 months during the 3 years post-surgery.

Additionally, the functionality of the affected limb was assessed every 2 months in the first 2 years following surgery, every 3 months in the third year, and every 6 months thereafter. Functional evaluation was conducted using MSTS scores, including six categorical scores for pain, function, emotional acceptance, use of supports, walking ability, and gait. These items were assessed with a 5-point scoring method with a maximum possible score of 30, according to the method provided by Enneking et al. [26]. Outcomes including immediate local complications (i.e., swelling and pain), recurrence (assessed by X-ray), and metastasis (determined by chest CT scanning) were reported along with the 3-year overall survival (OS) and disease-free (DFS) survival rates.

### Statistical analysis

All data were statistically analyzed using SPSS v.13.0 (SPSS, Inc., Chicago, IL, USA) and expressed as mean values ± standard deviation (SD), and categorical data were described as absolute frequencies. The 3-year OS rate, 3-year DFS rate, and local recurrence between patients with and without pathologic fracture were evaluated using χ^2^ tests or fisher’s exact tests. MSTS score comparison was completed using Student’s *t*-tests. All survival data, including both OS and DFS, were analyzed using the Kaplan-Meier method and a log-rank test. Statistical significance was defined as *P* values less than or equal to 0.05 (*P* ≤ 0.05).

## Results

### Demographic and clinical findings in patients with and without pathological fractures

Patients with pathologic fractures (n = 15) had a mean age of 23.2±8.0. Compared with the mean age of 21.8±12.2 in patients without pathologic fracture (n = 50), no significant difference was observed (*P* = 0.69). Patients with and without pathologic fractures exhibited stage IIB tumors with classic subtypes (14 *vs.* 49 patients, respectively) or other subtypes (1 *vs.* 1 patient, respectively), indicating no significant difference between these patient groups (*P* = 0.36). No significant differences were observed in tumor sites in patients with and without pathologic fractures, presenting in the femur (8 *vs.* 27 patients, respectively), tibia (2 *vs.* 7 patients, respectively), and humerus (5 *vs.* 16 patients, respectively) (*P* = 0.99) (Table 2).

### Oncologic outcomes in patients with pathological fractures

**Figure 1:**
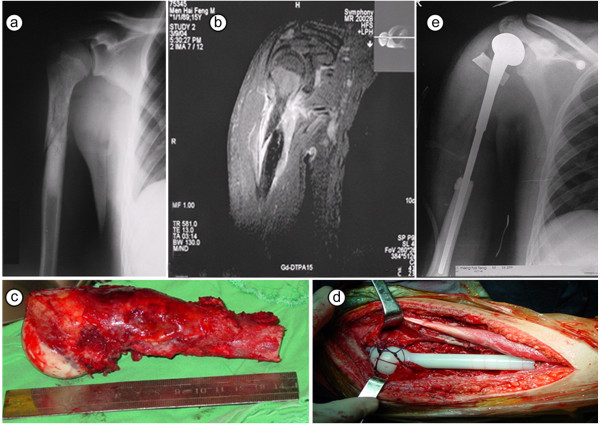
**Localized osteosarcoma complicated by pathologic fracture of the humerus in an 18-year old patient.** (**a**) X-ray of A-P position for patient at presentation; (**b**) Preoperative MRI indicating extraosseous lesion and undisplaced fracture of the humerus; (**c**) The resected specimen including the proximal humerus and biceps at the osteotomy level; (**d**) Reconstruction with semi-shoulder prosthesis; (**e**) Postoperative X-ray of affected arm.

Wide margin resections (as defined in Patients and methods [10,25]) were successfully achieved in 13 of 15 patients (86.7%) in the fracture group (5 amputation patients, 8 limb salvage surgery patients). The mean duration of surgery was 2.5 h (range, 1.5-3.5 h) and the mean intraoperative blood loss was 866 mL. Of these patients, local recurrence was observed in 3 of 10 limb salvage surgery patients and 1 of 5 amputation patients. Photographic and radiographic images of localized osteosarcoma complicated by pathologic fracture of the humerus in an 18-year old patient are shown in Figure 1.

### Complications associated with limb salvage surgery

Complications occurred in three limb salvage surgery patients. A 19-year-old boy presented with a loosened prosthesis 2 years after rotational hinged knee prosthesis reconstruction surgery, requiring revision surgery. A 22-year-old female suffered deep wound infection, requiring positive dressing and antibiotic administration for 3 months. In this case, poor wound healing resulted in secondary treatment by amputation. Femoral prosthesis breakage was observed in a 27-year old male 5 months after limb salvage and reconstruction surgery, requiring secondary prosthesis reconstruction surgery. This case resulted in death due to pulmonary metastasis 13 months after the second surgery. Additionally, four patients developed pulmonary metastasis leading to death (Table 3).

**Table 3:** Surgical data, MTST scores, and clinical outcomes of patients with or without pathologic fracture

	**Pathologic fracture patients (n = 15)**	**Non-fracture patients (n = 50)**	***P *value (χ**^**2 **^**test, Student's *t*-test)**
**Surgical technique**			
Amputation	5	4	
(Mean MTST score)	18.8±3.5	21.2±4.6	0.040
Limb salvage	10	46	
(Mean MTST score)	23.5±3.9	25.3±4.2	0.219
**Surgical margin**			
Pseudocapsule rupture	14 (93.3%)	5 (10.0%)	<0.001
Wide margin	13 (86.7%)	44 (88.0%)	0.890
Other margins	2 (13.3%)	6 (12.0%)	
**Outcomes**			
Recurrence	4 (26.7%)	7 (14.0%)	0.25
Complications	3 (20%)	6 (12.0%)	0.43
Metastasis	4 (26.7%)	16 (32.0%)	0.69
3-year DFS	53.3%	66.5%	0.25
3-year OS	66.7%	75.3%	0.52

MSTS: Musculoskeletal Tumor Society; DFS: Disease-free survival; OS: Overall survival.

### MSTS scores and functional outcomes

In the fracture group, the mean MSTS scores of limb salvage surgery patients were significantly higher compared to those of patients that underwent amputation (23.5 *vs.* 18.8, respectively) (*P* = 0.0002). Functional preservation was significantly better in limb salvage patients than patients that underwent amputation in both groups, with all surviving patients able to lead independent lives. Notably, five patients in the fracture group were able to partake in simple physical activities, such as jogging.

### Comparison of patients with and without pathologic fracture

**Figure 2:**
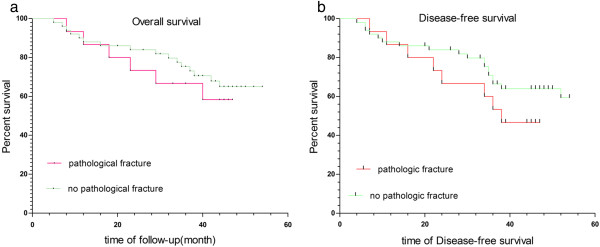
**Overall and disease-free survival percentage by month.** (**a**) Kaplan-Meier survival curve indicates no statistical difference in the overall survival rate between patients in the study and control groups; (**b**) Kaplan-Meier survival curve indicates no statistical difference in disease-free survival rate between patients in the study and control groups.

The 3-year OS rate for fractured patients was 66.7% *vs.* 75.3% in patients without pathological fracture. OS and DFS percentage by month are shown in Figure 2a and b. Patients without pathologic fracture showed relatively higher survival rates, but no significant difference were observed between patients with and without pathological fractures (*P* = 0.5190). The 3-year DFS rate for fracture group was 53.3% *vs.* 66.5% in the control group (*P* = 0.2466), and the local recurrence rate of the fracture group was 26.7% *vs.* 14% in the control group (Table 3, Clinical Outcomes). No significant variations were observed in the gender and age distribution in the fracture group.

In the fracture group, 14 of 15 patients (93%) avoided rupture of the tumor pseudocapsule, whereas only six cases (12%) were observed in the 50 patients of the control group (*P* = 0.000001). Wide surgical margins were achieved in 13 of 15 patients (86.7%) in the fracture group, *vs.* 44 of 50 patients (88%) in the control group (*P* = 0.890), with no statistical difference observed between groups. A total of 10 patients in the fracture group underwent limb salvage surgery, while only four patients failed of the control group were ineligible for limb salvage surgery (*P* = 0.039).

## Discussion

The current study details the experiences of a single hospital in management of patients with localized osteosarcoma complicated by pathologic fracture. We attempted to ascertain whether pathological fracture had prognostic significance or affected the efficacy of surgical limb salvage. Our study did not show significant differences between fracture and non-fracture patients for the outcome measures that were evaluated, namely recurrence, complications, metastasis, 3-year DFS, or 3-year OS. Therefore, we concluded, in our hospital setting, the presence of a pathologic fracture was not a prognostic indicator of recurrence or overall survival in localized osteosarcoma patients.

This study shows that a cooperative multidisciplinary approach involving orthopedic oncologists, medical oncologists, radiologists, pathologist, radiologists specializing in bone and soft tissue tumors, reconstructive surgeons, and physiotherapists, is required to effectively treat individual patients. Unfortunately, many medical facilities do not have access to this diverse set of clinicians, potentially limiting patient outcomes. Although several different postoperative complications were observed, all surviving patients exhibited good functionality at the final follow-up. When possible, limb salvage should be considered for these patients, as success rates of limb-preserving surgeries are dramatically higher that those observed only a few decades ago.

Successful limb salvage treatment should be considered in fractured patients with localized limb osteosarcoma that does not invade major neurovascular bundles or major regional muscles. In addition, the current study demonstrated that patients who respond well to preoperative chemotherapy without evidence of tumor skip or transfer may undergo limb-sparing treatment with minimal risk of metastasis. In order to further prevent such metastatic occurrences and prevent recurrence, wide surgical margins for complete resection of tumor are critical factors that affect the rate of local recurrence.

In the current study, certain limb salvage surgery cases presented unusual complications, such as femoral fatigue fracture and prosthetic loosening following reconstruction. It is well known that oncologic reconstruction involves higher complication rates than standard total joint arthroplasty [27]. This observation is primarily due to the extensive nature of the operation, involving significant tissue loss, and the compromising effect of associated chemotherapy. To mediate this relatively high rate of complications, risks and types of complications should be carefully assessed, particularly in physically active adolescent and young adult patients. Additionally, prosthesis construction should be carefully considered, as the present study showed that soft tissue transfer played an important role in both functional restoration and wound healing.

The prognostic importance of pathologic fractures in localized limb osteosarcoma and local disease control has been varied in the published literature. Ferguson et al. [12] compared oncological and functional outcomes of 201 patients with high-grade osteosarcoma without pathologic fracture to 31 patients with pathologic fractures, concluding that pathologic fracture was associated with decreased overall survival but not associated with local recurrence. These results were confirmed by subsequent studies [5,15]. In order to assess the value of pathological fracture as a prognostic indicator, the current study showed that clinical outcomes of limb salvage surgery and amputation resulted in different OS rates, DFS rates, and local recurrence. Consistent with the current study, numerous previous studies [4, 10, 14, 18], [23] have demonstrated that pathologic fractures, when treated with sufficient preoperative and postoperative chemotherapy and resected with adequate surgical margins, have no significant association with survival rate.

The current findings suggest that limb-sparing treatments can be successfully applied in many cases of localized limb osteosarcoma. In fact, limb salvage surgery produced significantly superior results in patients with and without pathological fractures, resulting in better functional outcomes. While limited in previous decades by the lack of sophisticate radiographic techniques and precision surgical tools, limb salvage surgery in contemporary clinical settings, coupled with modern chemotherapy, may be more effective than previously reported.

Tumor size has been shown to be an important risk factor for osteosarcoma patients [12]. However, tumor size measurements, conducted by MRI, may be inaccurate due to collapse or displacement that occurs secondary to fracture. Similar inaccuracies in tumor measurements may also occur in the presence of significant hematoma in the adjacent tissues [10,17]. To overcome this potential source of error, proximal location is sometimes more carefully considered than tumor size [15]. Bacci et al. [14] reported that humeral or diaphyseal location was an important risk factor in cases of pathologic fracture. Because radiographic imaging volumes were applied in the present study, tumor volumes may be somewhat inaccurate; however, this error was likely minimized in the absence of hematoma and tumor invasion.

## Conclusions

The current study examined patients receiving both limb salvage and amputation surgery. As suggested by Scully et al. [5], it reduces bias due to stratification by treatment strategy by reporting on both prognostic indicators and treatment strategies. The findings of the current study may, however, be limited by the assessment of tumor margin and pseudocapsule by the expert opinion of only a single surgeon and the relatively small number of included patients. This relatively small cohort precludes certain statistical analyses that would be interesting if demonstrated on a larger scale. Due to the rarity of the condition in the general population, obtaining a large cohort at a single center would be challenging, and future studies should consider combined multi-center analysis of these patients. A benefit of this study, however, is that the small patient number ensured that all included patients underwent follow-up until death or recurrence, with no exceptions. Further, unlike many retrospective studies, the experimental and control groups are matched for age, tumor stage, tumor site, and subtype, lending further credibility to the current findings.

Pathologic fracture was not shown to be a prognostic indicator for OS or DFS in localized primary osteosarcoma patients. Modern technology and imaging allows improved results in limb salvage surgery, producing satisfactory oncologic outcomes when adequate surgical margins are applied and combined with regular adjuvant chemotherapy. Unlike amputation surgery, limb-sparing treatments allow greater functional restoration and generally produce more positive overall patient outcomes. However, due to the risk of severe postoperative complications, patients and clinicians should carefully consider the risks associated with both amputation and limb salvage surgeries. While future study will be required before broad clinical recommendations can be made for treatment selection, the current study provides an important resource for future, evidence-based clinical studies.

## Abbreviations

DFS: Disease-free survival; MSTS: Musculoskeletal Tumor Society; OS: Overall survival.

## Competing interests

The authors declare that they have no competing interests.

## Authors’ contributions

HY, CZ carried out the studies, participated in collecting data, and drafted the manuscript. ZD, ZL performed the statistical analysis and participated in its design. SW helped to draft the manuscript. All authors read and approved the final manuscript.
